# Extraction Processes, Bioaccessibility, Antioxidant Capacity, and Potential Prebiotic Effect of Co‐Product Extracts From Fruits of the *Spondias* Genus

**DOI:** 10.1111/1750-3841.70260

**Published:** 2025-05-07

**Authors:** Ivania Samara dos Santos Silva Morais, Lucas Monteiro Bezerra Pinheiro, Fernanda Pereira Santos, Marcos dos Santos Lima, Karina Maria Olbrich dos Santos, Carolina Lima Cavalcanti de Albuquerque, Haissa Roberta Cardarelli

**Affiliations:** ^1^ Postgraduation Program in Food Science and Technology, Department of Food Engineering Federal University of Paraíba João Pessoa Brazil; ^2^ Department of Food Technology Federal Institute of Sertão de Pernambuco Petrolina Brazil; ^3^ Embrapa Food Agroindustry Brazilian Agricultural Research Corporation Rio de Janeiro Brazil; ^4^ Department of Food Technology, Center for Technology and Regional Development Federal University of Paraiba João Pessoa Brazil

**Keywords:** conventional extraction, prebiotic compounds, supercritical fluid extraction, total phenolic content, ultrasonic bath

## Abstract

This study evaluated which extraction methods among agitation (ethanol and water, 60 min), ultrasonic bath–assisted (ethanol and water, 15 min), and supercritical fluid extraction (CO₂ and ethanol, 40°C, 15 Mpa) would be superior for producing co‐product extracts from seriguela (*Spondias purpurea*), caja (*Spondias mombin*), and umbu‐caja (*Spondias* spp.). The bioaccessibility of phenolic compounds and potential prebiotic effects were also investigated. The in vitro prebiotic effect of the extracts was tested using *Lactobacillus acidophilus* (La‐3), *Bifidobacterium animalis* subsp. lactis (BB‐12), and *Lactiplantibacillus plantarum* (CNPC004) through cell viability and pH monitoring over 48 h, along with their prebiotic score against an enteric mixture (*Escherichia coli*). Ultrasonic bath–assisted extraction achieved the highest antioxidant capacity and total phenolic content across all extracts; in contrast, supercritical fluid extraction exhibited the lowest results, primarily for the seriguela extract (32.09 ± 0.89 mg GAE/100 g). Although the bioaccessibility of total phenolics and antioxidant capacity decreases after in vitro digestion, some individual phenolics exhibited high bioaccessibility levels, such as epicatechin gallate (135.5%) in caja extract and 125.3% in seriguela; catechin (106.6%) in seriguela; and gallic acid (108.5%) in umbu‐caja. All extracts positively influenced probiotic viability, with a 2‐log CFU/mL growth in all strains by the end of incubation. Seriguela extract showed the best results, with a final pH of 3.57 and higher cell counts, particularly for CNPC004 (9 log CFU/mL), and the highest prebiotic score among the co‐products. These findings indicate that ultrasound‐assisted extraction effectively captures phenolic compounds from *Spondias* co‐products, suggesting promising biological applications due to the bioaccessibility and prebiotic activity of the phenolic compounds.

**Practical Application**: Extracts from *Spondias* fruit co‐products offer innovative applications, combining antioxidant properties with potential prebiotic effects. These extracts can be utilized as functional ingredients in food products and nutraceuticals, highlighting their value in promoting health and well‐being.

## 1 Introduction

Industrial fruit production has been gaining prominence due to modernization and processing reaching a large scale, which has resulted in generating many co‐products that are usually underutilized or even inappropriately discarded. Reusing these co‐products can serve as a valuable source of nutrients, as they are reservoirs of compounds with functional properties such as fibers, polysaccharides, and bioactive compounds (Manai et al. 2024).

There are a wide variety of exotic fruit species in Brazil, especially in the Caatinga (the largest biome in the country) (Sviech et al. 2022), such as the fruits of the *Spondias* genus. The yellow‐orange, caja (*Spondias mombin*), seriguela (*Spondias purpurea*), and umbu‐caja (*Spondias* spp.) fruits have great acceptance among the genus due to their sensory characteristics, such as sweet and sour flavor, in addition to the presence of phytochemical compounds, such as phenolics (Cangussu et al. 2024), including catechin, quercetin, and chlorogenic acid (among others) (Á. G. F. Silva et al. 2025).

The phenolic compounds present in *Spondias* fruits exhibit various bioactive effects, including antioxidant (L. F. R. Oliveira, Medeiros, et al. 2024), antihypertensive (Macêdo et al. 2023), and neuroprotective activities (Ajayi et al. 2020). Furthermore, J. N. Oliveira, Albuquerque, et al. (2024) demonstrated that the phenolic compounds present in umbu‐caja flour played a significant role in inducing a prebiotic effect, thereby contributing to the positive modulation of the intestinal microbiota.

However, although promising, studies with species of the *Spondias* genus related to prebiotic activity are still scarce. Previous studies using other raw materials have already demonstrated that phenolic compounds exert prebiotic effects. Massa et al. (2020), Morais et al. (2019), Bonifácio‐Lopes et al. (2023), and Z. Liu et al. (2022) observed this effect in jabuticaba byproducts, red pitaya pulp, beer byproduct extract, and green and black tea, respectively.

Phenolic compounds, such as phenolic acids and flavonoids, possess a complex structure and are largely not absorbed in the small intestine, reaching the colon almost intact. Microorganisms such as *Lactobacillus* and *Bifidobacterium* activate specific biotransformation pathways to metabolize them. Microbial enzymes, such as β‐glucosidases, initially hydrolyze glycoconjugates, releasing the aglycones. Decarboxylation and dehydroxylation reactions subsequently occur, catalyzed by decarboxylases and dehydroxylases, which simplify the aromatic structure and lead to forming metabolites such as protocatechuic acid. In addition, side‐chain reduction and cleavage processes contribute to produce more stable and biologically active compounds. Phenolic compounds become more bioaccessible during this transformation and can be utilized by microorganisms as an alternative energy source, promoting their growth and acting as selective substrates for probiotic bacteria (J. Hu et al. 2024).

There are various ways of selecting and recovering these compounds, including through the extracts (Sanches et al. 2024). There are also various extraction methods which can be performed, such as agitation extraction (AE), constituting a more conventional approach, ultrasonic bath–assisted extraction (UBE), and supercritical fluid extraction (SFE). Agitation extraction relies on solubilization and mass transfer, where the sample is immersed in a solvent under continuous agitation. This enhances solvent penetration into the plant matrix, facilitating the release of phenolic compounds from cellular structures. However, AE often requires prolonged extraction times (Bonifácio‐Lopes et al. 2020).

UBE generates indirect ultrasonic radiation, creating cavitation bubbles which implode on plant tissue surfaces. This process enhances solvent penetration, disrupts cell walls, and promotes the release of phenolic compounds. Additionally, the formation and collapse of cavitation bubbles induce shear stress in the fluid, improving solute diffusion and extraction efficiency (Egüés et al. 2021).

SFE uses CO₂ in its supercritical state to dissolve target compounds while eliminating surface tension and enhancing diffusion through plant microchannels. Although CO₂ favors non‐polar compounds, the addition of polar co‐solvents like ethanol improves polyphenol extraction, increasing efficiency (Pilařová et al. 2024). Taking these factors into consideration, this study aimed to analyze the extraction of total phenolic compounds and antioxidant properties between agitation extraction, UBE, and supercritical fluid extraction methods. The study also investigated the bioaccessibility, antioxidant capacity of phenolic compounds, and potential prebiotic effect of the *Spondias* sp. fruit co‐product extracts in three types of probiotic bacteria: *Lactiplantibacillus plantarum* (CNPC004) *Lactobacillus acidophilus* (La‐3), and *Bifidobacterium animalis* subsp*. lactis* (BB‐12). Therefore, this study aims to demonstrate for the first time that in addition to their antioxidant properties, the phenolic compounds in umbu‐caja (UC), caja (CJ), and seriguela (SE) fruit co‐product extracts possess potential prebiotic effects, representing a novel contribution to applying phenolic compounds derived from fruit co‐products.

## 2 Materials and Methods

### 2.1 Raw Material

The residues from pulp processing SE, CJ, and UC were collected from agro‐industries during the harvest period (December to March) in the Caatinga region of Paraíba, where they were dried by convection drying in a forced air circulation oven (TECNAL, Piracicaba, SP, Brazil) at 40°C for 48 h. Afterward, the dry waste was crushed in a knife mill (Solab, Piracicaba, SP, Brazil), vacuum‐packed in polyethylene bags, and stored at room temperature.

#### 2.1.1 Agitation Extraction

AE was performed according to Silva Junior et al. (2021) with adaptations, using a mixture of ethanol (absolute 99.5°GL) (Sciavicco, Sabara, MG, Brazil), distilled water (55:45 v/v), and a raw material/solvent ratio of 1:40 (g/v). The sample and solvent were homogenized in Ultra‐Turrax (Sigma‐Aldrich, St. Louis, MO, USA) at 3500 rpm for 1 min. The mixture was agitated (200 rpm) for 60 min at a temperature of 22–24°C in an incubator with orbital agitation (Lucadema, Sao Jose do Rio Preto, SP, Brazil).

#### 2.1.2 Ultrasonic Bath‐Assisted Extraction

The same raw material/solvent ratio and ethanol‐water combination as the AE extraction were used for the UBE. The sample and solvent were homogenized in Ultra‐Turrax equal to AE. The Erlenmeyers with the mixture were placed in the ultrasonic bath (UNIQUE, Indaiatuba, SP, Brazil) and extracted for 15 min using 155 W of potency and 40 kHz, (22–24°C), according to the method described by Neto et al. (2022), with adaptations.

#### 2.1.3 Supercritical Fluid Extraction

The SFE System (Waters Corporation, Pittsburgh, PA, USA) was used for high‐pressure extraction, equipped with a thermostatic bath (Thermo Fisher Scientific, Waltham, MA, USA) maintained at −5°C, CO_2_ and cosolvent pumps, extraction vessel (500 mL), and two separator vessels (500 mL) for extract recovery, automatic “backpressure” regulating valves and a flow meter. The solvent was a mixture (10% m/m) of CO_2_ minimum purity of 99.9% (White Martins, João Pessoa, PB, Brazil) and ethanol, with a flow rate (flow of the solvent mixture passing through the system) of 20 g/min (18 g/min of CO_2_ + 2 g/min ethanol) and raw material/solvent ratio of 1:20 (g/v). The pressure and temperature used for the extractions were 15 MPa and 40°C (extraction vessel), 70 bar at 45°C (separator 1), and 40 bar at 40°C (separator 2), with these procedures based on Dias et al. (2019).

After completing the low‐pressure extractions (AE, UBE), the extracts were filtered on filter paper (Sigma‐Aldrich), while the SFE extract was collected in the device without solid residues.

All extracts were taken to a rotary evaporator (Biovera, Rio de Janeiro, RJ, Brazil) for ethanol evaporation using a pressure of 600 mbar and an average temperature of 40°C. The final volume was reconstituted with water distilled according to the initial volume, and the extracts were packed in amber glasses and stored in a freezer (−18°C).

### 2.2 Total Phenolic Content and Antioxidant Capacity

The total phenolic content (TPC) of the extracts was determined by the Folin–Ciocalteu method (Singleton and Rossi 1965). Antioxidant capacity was measured using the ferric reducing antioxidant power (FRAP), ABTS+ (3‐ethyl‐benzothiazoline‐6‐sulfonic acid), and DPPH (2,2‐diphenyl‐1‐picrylhydrazyl) assays, which are described by Benzie and Strains (1996), Re et al. (1999), and Brand‐Williams et al. (1995), respectively.

According to the results of the phenolic compounds and antioxidant capacity, the extract of each fruit residue that presented the best results was taken to the following stages.

### 2.3 Identification and Characterization of Phenolic Compounds

The profile of phenolic compounds was characterized by high‐performance liquid chromatography (HPLC) coupled with a diode array detector (DAD), following the methodology described by Padilha et al. (2017). An Eclipse Plus RP‐C18 column (100 × 4.6 mm, 3.5 µm) and Zorbax C18 pre‐column (12.6 × 4.6 mm, 5 µm) manufactured by Zorbax (Agilent Technologies, Santa Clara, CA, USA) were used. The separation was performed at 35°C with a 20‐µL injection volume and a flow rate of 0.8 mL/min. The mobile phase consisted of solvent A (0.52% phosphoric acid, pH 2.0) and solvent B (acidified methanol with 0.52% H3PO4), with a gradient from 5% B (0–5 min) to 80% B (30–33 min).

### 2.4 In Vitro Digestion Simulation

The in vitro digestion followed INFOGEST 2.0 (Mulet‐Cabero et al. 2020), simulating oral, gastric, and intestinal phases using respective fluids, enzymes (pepsin, pancreatin), and bile salts. Enzyme activity was halted between phases utilizing an ice bath. Two controls were included: one with water instead of samples and another without enzymes and bile. Aliquots were collected post‐digestion, centrifuged (3500 × g, 15 min), and stored at −20 ± 2°C for analysis.

#### 2.4.1 Bioaccessibility Index

The bioaccessibility index (BI%) of the phenolic compounds was calculated according to Vilas‐Boas et al. (2020), where the value of the compounds or antioxidant capacity before digestion is considered the initial value (100%) and the value of the compounds or antioxidant capacity after digestion is considered the final value, according to Equation (1):

(1) $$\mathrm{Bioaccessibility}\;\mathrm{index}\;\left(\%\right)=\frac{Bi_{\mathrm{PC}}}{Bi_{\mathrm{initial}}}\times 100,$$

where Bi_PC_ corresponds to the final duodenal value (mg/mL) and Bi_initial_ corresponds to the value of compounds/antioxidant capacity before digestion (mg/mL) of the extracts.

### 2.5 Potential Prebiotic Effect by Carbon Source Replacement and Prebiotic Score

The remaining extract from the intestinal phase (Duodenum) was placed in a semi‐permeable dialysis membrane, as described by T. M. R. Albuquerque et al. (2021). The final content was freeze‐dried and analyzed for prebiotic activity.

#### 2.5.1 Bacterial Strains and Inoculum Preparation

The strains were cultivated and standardized according to T. M. R. Albuquerque et al. (2020). Probiotic strains of *Lactiplantibacillus plantarum* (CNPC004), kindly provided by the reference collection of Embrapa, Brazil, *Lactobacillus acidophilus* (La‐3), and *Bifidobacterium animalis* subsp. *lactis* (BB‐12) were used for viability and prebiotic score evaluation. *Escherichia coli* strains (NewProv 0039 and ATCC11775), provided by the Laboratory of Biology of Microorganisms (BIOMICRO/UFPB, Brazil) and the Laboratory of Microbiology (CTDR/UFPB, Brazil), were used to prepare the enteric mixture inoculum (1:1) for indicating the prebiotic score. Glycerol stocks were grown twice in the Man, Rogosa and Sharpe (MRS) Kasvi, broth (CNPC004 and La‐3) for 24 h in anaerobiosis, except BB‐12 cultured in MRS broth supplemented with 0.5 g/L Cysteine‐L Hydrochoride (cMRS). They were centrifuged at 4500 × g for 10 min/4°C and resuspended in sterile saline solution to obtain standardized inoculum (OD of 0.8 at 655 nm).

*Escherichia coli* strains were used to prepare the enteric mixture inoculum for indicating the prebiotic score. The strains were grown in brain heart infusion (BHI; HiMedia, Mumbai, India) at 37°C for 24 h, centrifuged, washed, and resuspended (OD of 0.1 at 655 nm). The inoculum of the enteric mixture was the mixture (1:1) of the two strains.

#### 2.5.2 Preparation of the Cultivation Media

MRS and M9 (minimal medium) formulated broths were used as the base culture medium to evaluate the effect of digested extracts against probiotic and *E. coli* strains, respectively (Duarte et al. 2017). Different carbon sources (20 g/L) were analyzed to monitor the bacteria growth, including glucose (non‐prebiotic component); fructooligosaccharide (FOS) (prebiotic component); and the digested extracts of the co‐products of CJ, SE, and UC.

#### 2.5.3 Cell Enumeration and Viability

Each prepared inoculum was added to formulated broths (glucose, FOS, CJ, SE, UC) and then incubated at 37°C for 48 h. Aliquots were taken at different time intervals (0, 6, 12, 18, 24, and 48 h), followed by dilution and plating on cMRS agar for BB‐12, MRS agar for CNPC004 and La‐3, and methylene blue eosin agar (HiMedia) for the enteric mixture. BB‐12 culture media was incubated anaerobically. The viable cell count was expressed as log CFU/mL. The strains under study were also indirectly evaluated by measuring the pH values at the same time intervals during incubation using a digital potentiometer (Lucadema, Sao Jose do Rio Preto, SP, Brazil).

#### 2.5.4 Determination of Prebiotic Activity Scores

The selective growth of the probiotics induced by the extracts was verified by the quantitative score indicated by Equation (2) described by Huebner et al. (2007):

Prebiotic activity score = [(log CFU/mL of probiotic on the prebiotic at 24 h − log CFU/mL of probiotic on the prebiotic at 0 h)/(log CFU/mL of probiotic on glucose at 24 h − log CFU/mL of probiotic on glucose at 0 h)] − [(log CFU/mL of enteric mixture on prebiotic at 0 h)/(log CFU/mL of enteric mixture on glucose at 24 h − log CFU/mL of enteric mixture on glucose at 0 h)]. (2)

The prebiotic activity score is determined by comparing probiotic growth in an extract‐supplemented medium to growth in a glucose medium, considering enteric bacteria proliferation. A high score indicates selective promotion of probiotics, while a low or negative score suggests a lack of selectivity, favoring enteric bacteria.

### 2.6 Statistical Analysis

All assays were performed in triplicate, and the results were expressed as mean ± standard deviation. The data obtained in the analyses were evaluated by analysis of variance (ANOVA), followed by comparisons of means by Tukey's test at 5% significance using the Statistica 10.0 software (StatSoft, Tulsa, OK, USA).

## 3 Results and Discussion

### 3.1 Total Phenolic Compounds and Antioxidant Capacity of the Extracts

The TPC and antioxidant capacity of the UC, CJ, and SE extracts by different types of extraction are described in Table 1. The UBE method was the most efficient among all the analyzed methods (*p* < 0.05). This trend was also observed across all evaluated co‐products, presenting the best results for the SE and UC extracts, with 3377.00 ± 2.00 mg GAE/100 g and 3201.83±0.57 mg GAE/100 g, respectively. Differences were also found between agitation extraction and SFE, with SFE being the method that yielded the lowest results (*p* < 0.05). This behavior was consistent across all extracts. However, when only the SFE method was considered, the CJ extract (308.91 ± 0.92 mg GAE/100 g) exhibited higher results compared to UC (130.62 ± 0.92 mg GAE/100 g) and SE (32.09 ± 089 mg GAE/100 g) (*p* < 0.05). The TPC content by the SFE method was similar to Martins et al. (2022) when extracting phenolics by SFE from tamarind residues obtained 310 mg GAE/100 g.

**TABLE 1:** Antioxidant capacity and total phenolics (TPC) of umbu‐caja, caja, and seriguela co‐product extracts by different methods.

		Antioxidant methods			
Co‐products	Extraction methods	DPPH (µmol Trolox. g^−1^)	ABTS (µmol Trolox. g^−1^)	FRAP (µmol Trolox. g^−1^)	TPC (mg GAE.100 g^−1^)
Umbu‐caja	AE	51.21 ± 0.67b	123. 48 ±0.91b	106.59 ± 0.81b	2183.51 ± 1.03b
	UBE	75.06 ± 0.94a	168.72 ± 0.71a	154.02 ± 0.36a	3201.83 ± 0.57a
	SFE	5.11 ± 0.46c	39.03 ± 0.70c	32.91 ± 0.81c	130.63 ± 0.92c
Caja	AE	67.71 ± 0.54b	92.33 ± 0.57b	94.89 ± 0.74b	2015.00 ± 1.18b
	UBE	75.29 ± 0.13a	102.71 ± 0.27a	121.18 ± 0.54a	2539.55 ± 0.19a
	SFE	5.29 ± 0.00c	39.38 ± 0.03c	42.17 ± 0.01c	308.91 ± 0.79c
Seriguela	AE	223.29 ± 0.87b	85.36 ± 0.06b	130.18 ± 0.95b	3122.42 ± 1.15b
	UBE	236.49 ± 0.67a	175.23 ± 0.71a	186.67 ± 0.56a	3377.00 ± 2.00a
	SFE	0.15 ± 0.15c	0.19 ± 0.01c	<LOD	32.09 ± 0.89c

*Note*: Lowercase letters indicate a statistical difference between extraction methods, <LOD: below the limit of detection (LOD).

Abbreviations: AE, agitation extraction; SFE, supercritical fluid extraction; UBE, ultrasonic bath‐assisted extraction.

Dias et al. (2019) also compared phenolic extraction by the SFE and UBE methods in studies with *Spondias tuberosa* (umbu) seeds and obtained 25–35 mg GAE/100 g (SFE) and 76.0 mg GAE/g (UBE).

Extraction by the SFE method presents more satisfactory results in non‐polar molecules. By adding a co‐solvent such as ethanol, more polar compounds such as phenolics can be captured (Thai et al. 2024). However, it has not yet reached the levels of other methods for the extracts.

The efficiency of the UBE method compared to other methods has also been observed in other studies, such as in Fernandes et al. (2020) using jabuticaba bark, and in Silva Junior et al. (2021) using seriguela bark and obtaining 3515 mg GAE/100 g with UBE.

The antioxidant capacity of the extracts by the UBE method showed the best indices in all the raw materials analyzed (*p* < 0.05). The best result by the DPPH method was obtained in the SE extract with 236.49 ± 0.67 µmol TE/g, followed by the UC and CJ extract, which obtained 75.29 ± 0.13 µmol TE/g and 75.06 ± 0.94 µmol TE/g, respectively; this is higher than the antioxidant capacity found by Rufino et al. (2010) of 40.7 ± 2.2 µmol TE/g in the caja extract. The most satisfactory results for FRAP were also found in the SE extract by the UBE method, with 186.67 ± 0.56 µmol TE/g. The results of FRAP mirror those of the DPPH assay, as they exhibit similar mechanisms on single electron transfers (Pfukwa et al. 2022). The best results for ABTS were 168.72 ± 0.7 µmol TE/g, 102.71 ± 0.27 µmol TE/g, and 175.23 ± 0.71 µmol TE/g for the UC, CJ and SE extracts, respectively.

The antioxidant capacity results measured by DPPH and ABTS in guabiju bark extract and araça bark obtained through ultrasound‐assisted extraction also showed better responses among the analyzed methods (Bombana et al. 2023; Meregalli et al. 2020).

Carbon dioxide (CO_2_), used as a solvent, has non‐polar characteristics, which makes it difficult to solubilize intermediate or high polarity compounds, such as most phenolics, justifying the low antioxidant potential of the extracts obtained by this technique. Thus, different extraction processes can result in compounds with varying polarities (Dias et al. 2019), meaning that the interaction between antioxidants does not exclusively depend on the concentration of phenolics, but also on their structure and the synergism or antagonism between antioxidants (Ge et al. 2021).

The efficiency of UBE is related to its captive power through the microchannels created within the cells in the solid matrix that maximize solvent penetration, which improves mass transfer (Savic and Gajic 2020). In addition, this extraction method has other types of advantages when compared to traditional methods, as they can recover compounds with less extraction time, lower energy expenditure, and greater possibility of using organic solvents, such as ethanol and water (Ojha et al. 2020). Therefore, our results demonstrated the efficacy of the UBE technique in improving the recovery of phenolic compounds from UC, CJ, and SE co‐products. The UC, CJ, and SE extracts obtained by the UBE method were subsequently directed to the following analyses: phenolic compound profile, bioaccessibility, and investigation of potential prebiotic effects.

### 3.2 Bioaccessibility of Phenolic Compounds and Antioxidant Capacity After Simulated in Vitro Digestion

**FIGURE 1:**
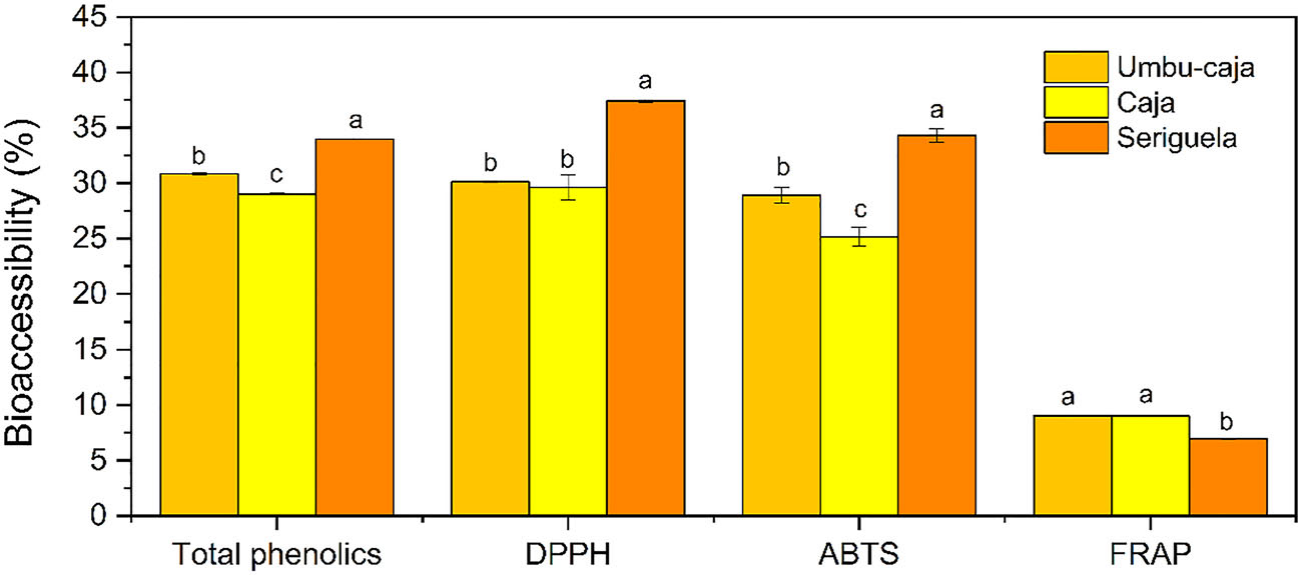
Bioaccessibility of total phenolics and antioxidant capacity of the umbu‐caja, caja, and seriguela co‐product extracts obtained by the ultrasonic bath‐assisted extraction method. Lowercase letters indicate statistical differences between raw materials.

The bioaccessibility index (Figure 1) is important to show which quantified compounds will probably be absorbed and exert functional action. Thus, it was possible to observe that the phenolics decreased in all extracts after digestion, and the best bioaccessibility level of TPC was found in seriguela with a utilization of 35%, followed by umbu‐caja with 31%, and caja, which presented the lowest level of bioaccessibility of TPC with 29%. Phenolic compounds can suffer instability during digestion due to pH conditions, which can lead to degradation, as well as other factors such as temperature, bile salts, and hydrolysis of enzymes that directly interfere with bioaccessibility (Y. Hu et al. 2023).

Many polyphenols are released during the gastric phase. However, TPC decreases upon reaching the intestinal phase where the pH is more alkaline due to the instability of small molecules formed in the gastric phase through the hydrolysis of more complex compounds (Stafussa et al. 2021).

Previous studies have also proven a significant decrease in TPC after in vitro digestion in beer byproduct extract, seriguela flour (Bonifácio‐Lopes et al. 2023; Cangussu et al. 2024). TPC can change the molecular structure or be biotransformed, generating other types of compounds (Tu et al. 2021).

The results show that the digestion also negatively affected the antioxidant capacity of the extracts. The best indexes for the DPPH and ABTS methods appeared in the SE extract, respectively, 37.25% and 35% antioxidant capacity. There was no statistical difference between CJ and UC extracts regarding FRAP, although it was inferior in the SE extract. Wu et al. (2022) and Pinto et al. (2024) also found lower antioxidant capacity after the digestion of fruit extracts.

Several factors can influence antioxidant capacity during the digestive process. Variations in pH throughout the gastrointestinal tract directly affect their stability and may induce degradation, oxidation, or polymerization processes, compromising their antioxidant efficacy. Furthermore, digestive enzyme action can alter the chemical structure of these compounds, modifying their biological activity. Hydrophilic compounds, such as phenolic acids and certain glycosylated flavonoids, are more susceptible to degradation in the intestinal environment due to their high water solubility. The reduction of these compounds during digestion may diminish the overall antioxidant capacity of the extract, thereby impacting its biological functionality (Canalis et al. 2020).

The methodological principles for measuring antioxidant capacity can influence the results (Pinto et al. 2024), as well as the molecular weight of the extracted compounds, and the type of molecules present (such as pigments, fibers) can directly influence bioaccessibility (Y. Hu et al. 2023). Therefore, this may explain the difference in results between ABTS and DPPH compared to FRAP. The antioxidant capacity and the TPC generally agree, which reinforces the role of the TPC in the antioxidant action of the extract (Zhang et al. 2023).

### 3.3 Profile of the Extract Phenolic Compounds During in Vitro Digestion and Its Bioaccessibility

**FIGURE 2:**
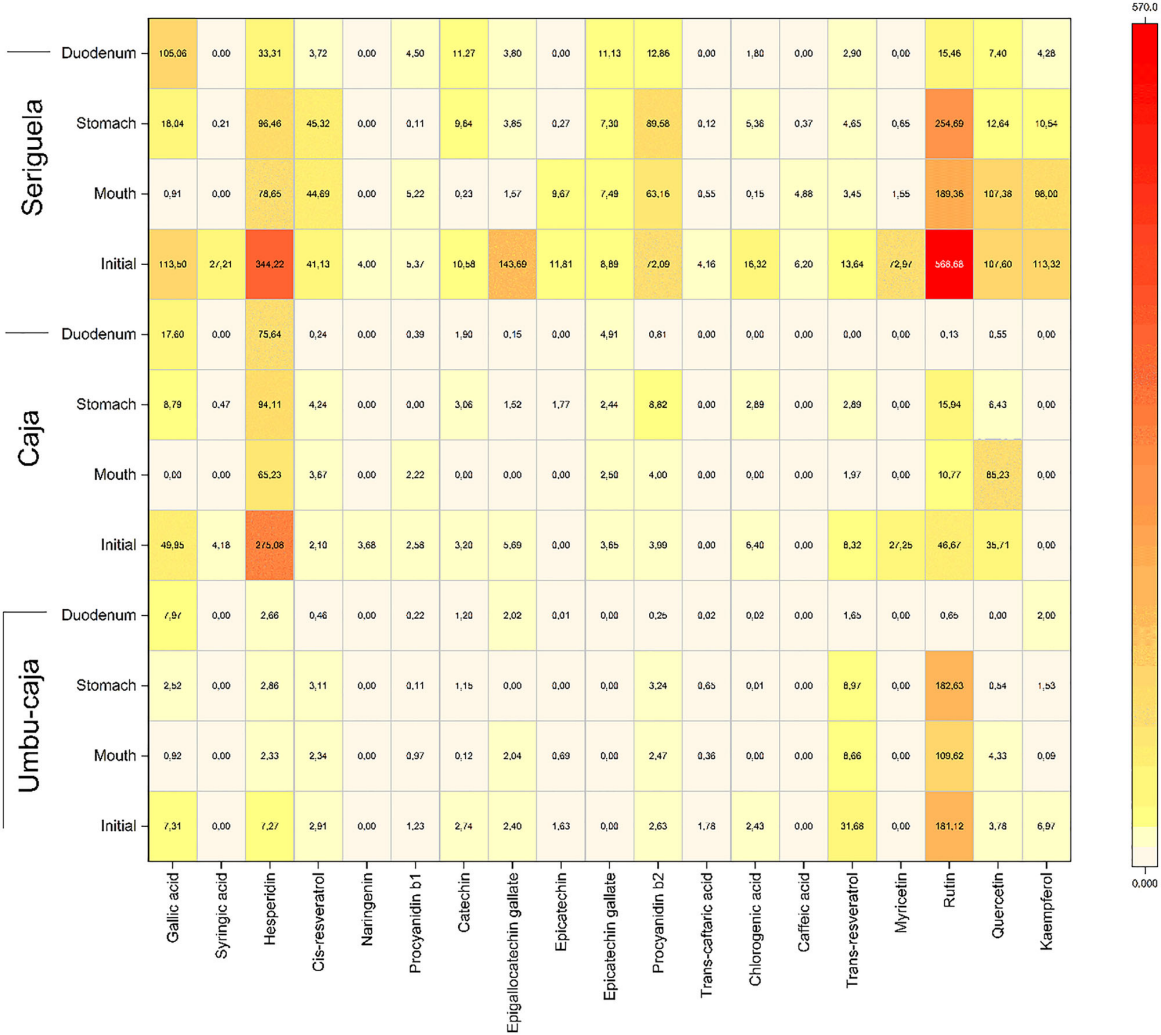
Phenolic profile of seriguela, caja, and umbu‐caja co‐product extracts during the digestion phases.

As they belong to the same genus (*Spondias*), the profile of phenolic compounds in the extracts (UC, CJ and SE) includes several compounds in common, such as gallic acid, hesperidin, cis‐resveratrol, procyanidin B1, catechin, epigallocatechin gallate, epicatechin, procyanidin B2, chlorogenic acid, trans‐resveratrol, rutin, and quercetin, as demonstrated in Figure 2.

SE extract presented higher quantifications of the isolated compounds than the CJ and UC extracts (Figure 2 and Table 2), while the compound with the highest initial fraction was rutin with about (568.70 mg/100 g), followed by hesperidin (344.22 mg/100 g), epigallocatechin gallate (143.69 mg/100 g), and gallic acid (113.50 mg/100 g). As is known, extraction processes can favor and disfavor some phenolic acids; however, rutin was also the predominant flavonoid when comparing the SE extract with the SE pulp in the study by Dutra et al. (2017). The profile of phenolics in the extracts is also in agreement with other studies that analyzed seriguela peel and flour from its residues in which compounds such as gallic acid, chlorogenic acid, quercetin, and kaempferol were detected (Cangussu et al. 2024; R. V. Silva et al. 2016).

**TABLE 2:** Profile and bioaccessibility of phenolic compounds from umbu‐caja, caja, and seriguela co‐product extracts.

Compounds (mg.100 g^−1^)	–	Umbu‐caja			Caja				Seriguela			
		Stage			Stage				Stage			
	Initial	Mouth	Stomach	Duodenum	Initial	Mouth	Stomach	Duodenum	Initial	Mouth	Stomach	Duodenum
Gallic acid	7.3 ± 0.0b	0.9 ± 0.0d	2.5 ± 0.1c	8.0 ± 0.0a	49.9 ± 0.0a	–	8.8 ± 0.0c	17.6 ± 0.0b	113.5 ± 0.4a	0.9 ± 0.0d	18.0 ± 0.1c	105.1 ± 0.0b
	% B: 108.9							%B: 35.3				%B: 92.6
Syringic acid	–	–	–	–	4.2 ± 0.0a	–	0.5 ± 0.0b	–	27.2 ± 0.0a	–	0.2 ± 0.0b	–
Hesperidin	7.3 ± 0.0a	2.3 ± 0.0d	2.9 ± 0.0b	2.7 ± 0.0c	275.1 ± 0.0a	65.2 ± 0.1d	94.1 ± 0.3b	75.6 ± 0.0c	344.2 ± 0.1a	78.6 ± 0.0c	96.5 ± 0.1b	33.3 ± 0.0d
	% B: 36.4							%B: 27.5				%B: 9.7
Cis‐resveratrol	2.9 ± 0.1b	2.3 ± 0.0c	3.1 ± 0.0a	0.5 ± 0.0d	2.1 ± 0.0c	3.7 ± 0.0b	4.2 ± 0.1a	0.2 ± 0.0d	41.1 ± 0.0c	44.7 ± 0.0b	45.3 ± 0.0a	3.7 ± 0.0d
	% B: 15.5							%B: 11.3				%B: 9.0
Naringenin	–	–	–	–	3.7 ± 0.0a	–	–	–	4.0 ± 0.0	–	–	–
Procyanidin B1	1.3 ± 0.0a	1.0 ± 0.0b	0.1 ± 0.0c	0.2 ± 0.0d	2.6 ± 0.0a	2.2 ± 0.0b	–	0.4 ± 0.0c	5.4 ± 0.0a	5.2 ± 0.0b	0.1 ± 0.0d	4.50 ± 0.0c
				% B: 17.19				%B: 15.1				%B: 83.7
Catechin	2.8 ± 0.1a	0.1 ± 0.0c	1.1 ± 0.0b	1.2 ± 0.0b	3.2 ± 0.0a	–	3.1 ± 0.1a	1.9 ± 0.0b	10.6 ± 0.0b	0.2 ± 0.0d	9.6 ± 0.0c	11.27 ± 0.0a
				%B: 43.0				%B: 59.1				%B: 106.6
Epigallocatechin gallate	2.4 ± 0.0a	2.0 ± 0.0b	–	2.0 ± 0.0b	5.7 ± 0.0a	–	1.5 ± 0.0b	0.1 ± 0.0c	143.7 ± 0.1a	1.6 ± 0.0c	3.8 ± 0.0b	3.8 ± 0.0b
				% B: 84.4				%B: 2.5				%B: 2.6
Epicatechin	1.6 ± 0.1a	0.7 ± 0.0b	–	–	–	–	1.8 ± 0.0a	–	11.8 ± 0.0a	9.7 ± 0.0b	0.3 ± 0.0c	–
Epicatechin gallate	–	–	–	–	3.6 ± 0.0b	2.5 ± 0.0c	2.4 ± 0.0c	4.9 ± 0.1a	8.9 ± 0.0b	7.5 ± 0.0c	7.3 ± 0.0d	11.13 ± 0.0a
								%B: 135.5				%B: 125.3
Procyanidin B2	2.6 ± 0.1b	2.5 ± 0.0b	3.2 ± 0.0a	0.2 ± 0.0c	4.0 ± 0.0b	4.0 ± 0.0b	8.8 ± 0.0a	0.8 ± 0.0c	72.1 ± 0.1b	63.1 ± 0.0c	89.6 ± 0.0a	12.9 ± 0.1d
				%B: 9.5				% B: 20.3				%B: 17.8
Trans‐caftaric acid	1.8 ± 0.0a	0.3 ± 0.0c	0.6 ± 0.0b	–	–	–	–	–	4.1 ± 0.0a	0.5 ± 0.0b	0.1 ± 0.0c	–
Chlorogenic acid	2.4 ± 0.0	–	–	–	6.4 ± 0.0a	–	2.1 ± 0.0b	–	16.3 ± 0.0a	0.1 ± 0.0d	5.4 ± 0.0b	1.8 ± 0.0c
												%B: 11.0
Caffeic acid	–	–	–	–	–	–	–	–	6.2 ± 0.0a	4.9 ± 0.0b	0.4 ± 0.0c	–
Trans‐resveratrol	31.7 ± 0.0a	8.7 ± 0.0c	8.9 ± 0.0b	1.6 ± 0.0d	8.3 ± 0.0a	2.0 ± 0.0c	2.9 ± 0.0b	–	13.6 ± 0.0a	3.4 ± 0.0c	4.6 ± 0.0b	2.9 ± 0.0d
				%B: 5.2								%B: 21.3
Myricetin	–	–	–	–	–	–	–	–	73.0 ± 0.0a	1.5 ± 0.0b	0.6 ± 0.0c	–
Rutin	181.1 ± 0.6 a	109.6 ± 0.0c	182.6 ± 0.0b	0.6 ± 0.0d	46.7 ± 0.1a	10.8 ± 0.1c	16.0 ± 0.0b	0.1 ± 0.0d	568.7 ± 0.0a	189.4 ± 0.0c	254.7 ± 0.0b	15.5 ± 0.0d
				%B: 0.36				%B: 0.27				%B: 2.7
Quercetin	3.8 ± 0.1a	4.3 ± 0.0b	0.5 ± 0.0c	–	35.7 ± 0.1b	85.2 ± 0.3a	6.4 ± 0.0c	0.5 ± 0.0d	107.6 ± 0.4a	107.4 ± 0.0a	12.6 ± 0.0b	7.4 ± 0.0c
									%B: 1.54			%B: 6.9
Kaempferol	7.0 ± 0.1a	0.1 ± 0.0d	1.5 ± 0.0c	2.0 ± 0.0b	–	–	–	–	113.3 ± 0.3a	98.0 ± 0.0b	10.5 ± 0.0c	4.3 ± 0.0d
				%B: 28.8								%B: 3.8

*Note*: Lowercase letters in each line indicate statistical differences (*p* < 0.05) between the stages of simulated gastrointestinal digestion. B% refers to the bioaccessibility of the individual compounds.

**TABLE 3:** Prebiotic activity scores (mean ± standard deviation) of fructooligosaccharides (FOS) (positive control) and the SE, CJ and UC co‐product extracts.

Probiotic	FOS	Seriguela	Caja	Umbu‐caja
BB‐12	0.98 ± 0.00aA	0.70 ± 0.11bB	0.85 ± 0.02abA	0.74 ± 0.02bB
CNPC004	1.22 ± 0.04aA	1.13 ± 0.09aA	0.56 ± 0.06bB	1.09 ± 0.01aA
La‐3	0.98 ± 0.18aA	0.67 ± 0.02abB	0.44 ± 0.03bB	0.62 ± 0.05abC

*Note*: Lowercase letters in each line indicate statistical differences (*p* < 0.05) between the FOS, umbu‐caja, caja, and seriguela media. Uppercase letters in each column indicate statistical differences (*p* < 0.05) between the probiotic BB‐12, CNPC004, and La‐3 strains.

The compounds with the highest occurrence concerning the CJ extract were hesperidin (275.08 mg/100 g), gallic acid (49.95 mg/100 g), rutin (46.67 mg/100 g), and quercetin (35.71 mg/100 g). Hesperidin is one of the main flavonoids present in fruits with citric characteristics, as in the case of fruits of the *Spondias* genus; this flavonoid presents antioxidant and anti‐inflammatory action (Gur et al. 2022).

T. C. S. Albuquerque et al. (2022) analyzed the profile of caja bagasse extract, and compounds such as rutin and gallic acid were also abundant. However, the phenolic profile can vary depending on the part of the fruit analyzed and other factors such as the ripeness stage and endoclimatic conditions (Cangussu et al. 2024).

The most representative compound in the UC extract was rutin (181.12 mg/100 g), the highest among the analyzed extracts, followed by trans‐resveratrol (31.68 mg/100 g). Rutin was also found in abundance in the study by Dutra et al. (2017) in UC pulp. It is worth noting that this is the first study that reports phenolic compounds from a UC residue extract.

Phenolic compounds undergo various structural transformations during simulation, leading to changes in their concentration across different phases. Factors such as pH and enzymatic activity can induce chemical modifications in the gastric phase, resulting in the conversion or degradation of these compounds (C. X. Li et al. 2023). Hesperidin exhibited higher concentrations in the gastric phase across all extracts throughout the digestive process, likely due to increased solubilization facilitated by the acidic environment (Hou et al. 2019).

It is possible to better observe how the compounds behaved during the simulated digestion and their respective bioaccessibility (%) in Table 2, which corresponds to the percentage of the compound released after the digestion process, being available for intestinal absorption and possible biological action (Pinto et al. 2023).

Epicatechin gallate showed the best bioaccessibility results with 135.5% for CJ extract and 125.3% for SE extract. This isolated phenolic is little studied, but some results show antioxidant action, anticancer properties, and a link to prebiotic effects (Z. Li et al. 2022; Z. Liu et al. 2022).

Some acids can reestablish themselves or even improve their bioavailability between gastrointestinal phases, which is what happened with gallic acid and catechin in all extracts, presenting a lower fraction in the oral phase, increasing in the stomach phase, and reaching its apex in the duodenal phase, except for CJ extract which presented a higher fraction of catechin in the stomach phase. Thus, the best bioaccessibility of catechin occurred in the SE extract in the duodenal phase with about 106.6%. The increase in catechin concentration may be related to the hydrolysis of procyanidin B1, which showed higher concentrations before digestion, as catechin is one of the components that forms procyanidins (X. Liu et al. 2021).

The gallic acid concentrations reached 108%, in SE and 92.6% in UC, respectively, representing some of the best results. The increase in gallic acid was also demonstrated after gastrointestinal simulation in the pulp of umbu‐caja (Dutra et al. 2017). The high bioaccessibility of these compounds may be caused by the partial hydrolysis of some flavonoids with polymeric structures, such as catechin, when exposed to intestinal pH conditions (Mosele et al. 2016).

Increased intestinal bioaccessibility of phenolic compounds can be explained by several factors, including the release of conjugated forms, enhanced solubilization, and interactions with extract components. For instance, gallic acid in CJ and UC extracts may have become more bioaccessible in the intestine due to the hydrolysis of tannins (Dutra et al. 2023). Other factors inherent to the digestive process can also influence the transformation of compounds during digestion, such as the solvent used in extraction (Kashyap et al. 2022).

Despite its high amount in the initial portion of all extracts, the rutin fractions were decreasing from the oral phase, with a slight recovery in the stomach phase and a huge loss when it reached the intestine, presenting only 0.36% in UC, 0.27% in CJ, and 2.7% in SE extracts. However, Dutra et al. (2017) reported better biaccessibility of rutin for umbu‐caja pulp (21%) and seriguela (17%).

The difference between these results may be the matrix in which the compound is present. The extracts are in liquid form, with the phenolic compounds mostly in their free form. Also, a small portion is bound to sugars or soluble fibers, making these compounds more susceptible to stressors (Vilas‐Boas et al. 2020).

The bioaccessibility of some flavonoids can be low and may vary according to composition, molecular weight, subclass, esterification, and glycosylation (T. Y. Wang et al. 2018). Thus, the flavonoids of the flavonol class (myricetin, rutin, quercetin, kaempferol) showed high sensitivity to digestive processes. This phenomenon may have occurred due to the low stability of compounds to pH variations during digestion, such as in quercetin (Cangussu et al. 2024). Zhao et al. (2023) also demonstrated the low bioaccessibility of myricetin during digestion.

The enzymatic action, pH, and solubilization of the duodenum fluids can release some low molecular weight compounds during the digestive process which are partially absorbed. The high molecular weight compounds will reach the colon where they can be retained in the indigestible fraction and be biotransformed by the microbiota, releasing aglycones and metabolites that can have bioactive properties (Pinto et al. 2023).

Given the accessibility and composition of phenolic compounds, the extracts show great potential for developing health‐promoting formulations for the pharmaceutical industry and as ingredients for food product additions.

### 3.4 Potential Prebiotic Effect by Carbon Source Replacement

To the best of our knowledge, this is the first study to evaluate the prebiotic capacity of *Spondias* extracts on probiotics *Lactobacillus plantarum* (CNPC004), *Lactobacillus acidophilus* (La‐3), and *Bifidobacterium animalis* subsp. *lactis* (BB‐12).

**FIGURE 3:**
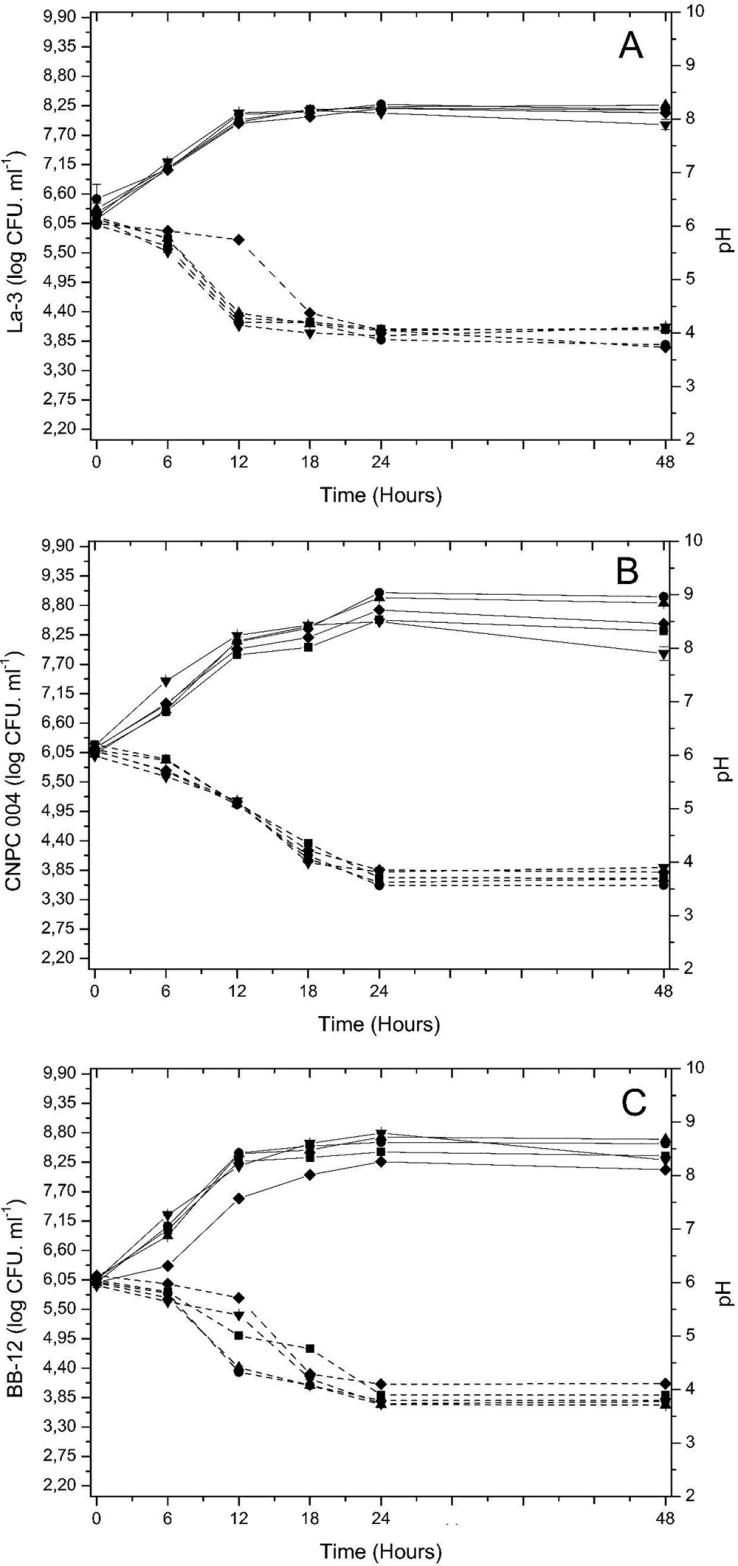
Cell viability and pH, for *L. acidophillus* La‐3 (A), *L. plantarum* CNPC004 (B), and *Bifidobacterium animalis* BB‐12 (C) in broth with glucose (▼), fructooligosaccharides (▲), caja extract (∎), seriguela extract (●), and umbu‐caja extract (◆) during 48 h of incubation at 37°C.

After the gastrointestinal simulated digestion, the prebiotic effect generated by UC, CJ, and SE extracts was evaluated on the probiotic La3, CNPC004, and BB‐12 strains. Figure 3A–C shows that none of the extracts showed a negative effect on the growth of the strains, with counts above 8 log CFU/mL after 48 h of incubation in SE, CJ, UC, and fructooligosaccharides (FOS) media, except for the glucose medium achieving less than 8 log CFU/mL. The viable cell counts increased by about 2 log until the end of the incubation.

The exponential growth duration of La‐3 in all media was up to 12 h, with no statistical difference between the samples (*p* < 0.05) (Figure 3A). Cell counts were higher after 24 h in SE, FOS, and CJ, with no statistical difference between the media cited. The broth with CJ, SE, and UC did not show a statistical difference at the end of the incubation time, reaching the range of 8.11–8.17 log CFU/mL, which was lower than the broth with FOS (*p* < 0.05), which presented 8.26 log CFU/mL. Sajib et al. (2018) and Bonifácio‐Lopes et al. (2023) also demonstrated that *Lactobacillus* strains used FOS medium more efficiently.

The data regarding the pH corroborate the cell viability, where the lowest pH occurred between 24 and 48 h, concomitant with the increase in the cell viability found in the medium with SE (pH around 3.7–3.8). Thus, the quantification of viable cells and the pH measured simultaneously demonstrate that the extracts served as substrates for the probiotics like the FOS standard, probably due to the fermentation of the components present in the extracts, particularly the phenolics.

The mechanism by which phenolic compounds exert a prebiotic effect has not yet been fully elucidated. However, certain phenolics, such as catechin, are known to be metabolized by probiotic bacteria. Catechin exhibited high bioaccessibility in the extracts, particularly in the SE extract. This compound may have contributed to the prebiotic effect observed, as the SE extract demonstrated the highest increase in cell viability (Barbosa et al. 2022).

Figure 3B illustrates the cell viability and pH of the CNPC004 strain between the extracts. As expected, the medium with glucose was more efficient as a carbon source for the *Lactobacillus* strain at the beginning of incubation. However, after 24 h, it showed a considerable decrease in cell viability.

Melo et al. (2020) demonstrated that glucose consumption was depleted in the broth after 24 h of fermentation, possibly consumed by the high metabolic active strains. It is already recognized that microbial species prefer simple carbohydrates at first, such as glucose, due to the ease of metabolization and consequent conversion into byproducts of metabolism, such as lactate (Sousa et al. 2015). Similar behavior was observed in the study by Spréa et al. (2024).

The broth containing SE showed the best results after 24 h of cultivation (*p* > 0.05), achieving the highest viable cells compared to the other probiotics, reaching 9 log CFU/mL. *L. plantarum* shows overexpression of genes that help in the transcriptomic response to tannins, such as gallic acid, one of the phenolics most found in the extracts, which connotes better adaptation of microorganisms to tannin‐rich environments (Reverón et al. 2013). These metabolic characteristics demonstrate that *L. plantarum* is a promising candidate for phenolic‐linked synbiotics (Rodríguez‐Daza et al. 2021), and the tested CNPC004 *L. plantarum* strain has such potential when growing in SE medium.

The cell viability of BB‐12 can be observed in Figure 3C. The media with FOS and glucose did not differ statistically at 24 h, showing 8.72 and 8.79 log CFU/mL, respectively, and achieving the highest viability results (*p* < 0.05). The cell viability of the medium with FOS stood out during incubation, which may be related to the polymerization degree being more favorable for *Bifidobacteria* metabolism (Scott et al. 2020).

Among the media with extracts, the broth with SE demonstrated similar behavior to the FOS standard, showing greater viability at all times compared with UC and CJ. The broth with UC presented the lowest viability with the probiotic BB‐12 (*p* > 0.05) among the extracts from 6 h of fermentation. All broths showed a decline in viability at the end of incubation (*p* < 0.05), as occurred in the studies by Alves‐Santos et al. (2023) when analyzing the prebiotic activity of Baru in *Bifidobacteria* and beer extract in Bonifácio‐Lopes et al. (2023).

The 48‐hour cultivation of the probiotic bacteria in the broths reduced the initial pH values in all growth media. The decrease in pH values is due to the organic acids, which are co‐products of fermentation (Gibson et al. 2017). The lowest pH (3.56) was found in the broth with SE for CNPC004 within 48 h of incubation.

The extracts mainly contain phenolic compounds, which may have stimulated the viability of probiotics. After microbial deglycosylation, phenolics can serve as carbon sources, and some acids, such as gallic acid, can also generate a proton‐driving force (Rodríguez‐Daza et al. 2021). In addition, other phenolics found in the extract and with good bioaccessibility, such as procyanidins, epicatechin gallate, and epicatechin, have been shown to stimulate the growth of *Bifidobacterium* spp. and *Lactobacillus* species (Souza et al. 2019).

These probiotic bacteria metabolize phenolic compounds through biotransformation. The process is mediated by microbial enzymes, such as glycosidases, which break glycosidic bonds to release bioactive aglycones; esterases, which hydrolyze ester bonds, converting compounds like chlorogenic acid into caffeic acid; and decarboxylases, which cleave carboxyl groups, generating smaller metabolites and increasing bioavailability (Tang et al. 2022). The findings highlight the potential applications of the extracts in functional foods and dietary supplements, where it could contribute to gut health by enhancing microbial balance.

#### 3.4.1 Prebiotic Activity Score

Table 3 shows the results of the prebiotic activity scores. All the co‐products analyzed obtained positive prebiotic activity scores for the probiotics analyzed with the enteric mixture. The best results were in CNPC004, with no statistical difference between broths with FOS, SE, and UC. The highest prebiotic activity scores for BB‐12 and La‐3 between the extracts were those with CJ (0.85 ± 0.02) and SE (0.67 ± 0.02). Given the definition by Gibson et al. (2017), for a component to be considered a prebiotic, it should resist digestion in the host, be fermentable by intestinal microorganisms, and be capable of stimulating the selective growth of beneficial bacteria of the microbiota. Based on this principle, the prebiotic activity scores of SE, CJ, and UC digested extracts indicate their ability to stimulate the selective growth of *L. plantarum*, *Lactobacillus acidophilus*, and *Bifidobacterium animalis* subsp. *lactis* compared to enteric pathogens (Duarte et al. 2017; S. Wang et al. 2020).

## 4 Conclusion

The UBE method proved most effective for the seriguela, caja, and umbu‐caja co‐products, with significantly higher total phenolic compounds and antioxidant capacity than in the extracts obtained by SFE and the agitation method. This efficiency resulting from cavitation induced by ultrasonic waves highlights the potential of UBE to enhance phenolic compound recovery in fruit co‐products. The phenolics in the extracts were bioaccessible and exhibited post‐digestion antioxidant capacity. Some phenolics, such as gallic acid in UC and SE, catechin in SE, epicatechin gallate in CJ and SE, and procyanidin B1 in SE, were more bioaccessible. All extracts induced the selective growth of the tested probiotics, especially the CNPC004 strain which presented the highest number of viable cells. The SE extract showed better effects on the growth of the strains, which also stood out for the total phenolic content and antioxidant capacity, both at the beginning and after digestion. Therefore, the results suggest a potential relationship between the phenolic content and its role as a selective substrate for probiotic bacteria, contributing to the prebiotic effect observed in the extracts. Additional studies should evaluate which phenolic compounds are most consumed by probiotic bacteria and analyze the effect of fermented extracts on fecal microbiota, providing insights and bases for using extracts as prebiotic additives.

## Author Contributions

**Ivania Samara dos Santos Silva Morais**: Conceptualization, methodology, investigation, validation, writing–original draft, formal analysis. **Lucas Monteiro Bezerra Pinheiro**: Investigation, formal analysis. **Fernanda Pereira Santos**: Investigation, formal analysis. **Marcos dos Santos Lima**: Investigation, formal analysis. **Karina Maria Olbrich dos Santos**: Investigation, formal analysis. **Carolina Lima Cavalcanti de Albuquerque**: Methodology, formal analysis, writing–review and editing, supervision. **Haissa Roberta Cardarelli**: Conceptualization, writing–review and editing, supervision, validation, resources.

## Conflicts of Interest

We declare no conflicts of interest.
